# Improved Air Quality and Attenuated Lung Function Decline: Modification by Obesity in the SAPALDIA Cohort

**DOI:** 10.1289/ehp.1206145

**Published:** 2013-07-02

**Authors:** Tamara Schikowski, Emmanuel Schaffner, Flurina Meier, Harish C. Phuleria, Andrea Vierkötter, Christian Schindler, Susi Kriemler, Elisabeth Zemp, Ursula Krämer, Pierre-Olivier Bridevaux, Thierry Rochat, Joel Schwartz, Nino Künzli, Nicole Probst-Hensch

**Affiliations:** 1 Swiss Tropical and Public Health Institute, Basel, Switzerland; 2 University of Basel, Basel, Switzerland; 3 Department of Epidemiology, Leibnitz Institut for Environmental Medicine at the Heinrich-Heine University (IUF), Düsseldorf, Germany; 4 Division of Pulmonary Medicine, University Hospitals of Geneva, Geneva, Switzerland; 5 Department of Environmental Health, Harvard School of Public Health, Boston, Massachusetts, USA

## Abstract

Background: Air pollution and obesity are hypothesized to contribute to accelerated decline in lung function with age through their inflammatory properties.

Objective: We investigated whether the previously reported association between improved air quality and lung health in the population-based SAPALDIA cohort is modified by obesity.

Methods: We used adjusted mixed-model analyses to estimate the association of average body mass index (BMI) and changes in particulate matter with aerodynamic diameter ≤ 10 µm (PM_10_; ΔPM_10_) with lung function decline over a 10-year follow-up period.

Results: Lung function data and complete information were available for 4,664 participants. Age-related declines in lung function among participants with high average BMI were more rapid for FVC (forced vital capacity), but slower for FEV_1_/FVC (forced expiratory volume in 1 sec/FVC) and FEF_25–75_ (forced expiratory flow at 25–75%) than declines among those with low or normal average BMI. Improved air quality was associated with attenuated reductions in FEV_1_/FVC, FEF_25–75_, and FEF_25–75_/FVC over time among low- and normal-BMI participants, but not overweight or obese participants. The attenuation was most pronounced for ΔFEF_25–75_/FVC (30% and 22% attenuation in association with a 10-μg/m^3^ decrease in PM_10_ among low- and normal-weight participants, respectively.)

Conclusion: Our results point to the importance of considering health effects of air pollution exposure and obesity in parallel. Further research must address the mechanisms underlying the observed interaction.

Citation: Schikowski T, Schaffner E, Meier F, Phuleria HC, Vierkötter A, Schindler C, Kriemler S, Zemp E, Krämer U, Bridevaux P-O, Rochat T, Schwartz J, Künzli N, Probst-Hensch N. 2013. Improved air quality and attenuated lung function decline: modification by obesity in the SAPALDIA cohort. Environ Health Perspect 121:1034–1039; http://dx.doi.org/10.1289/ehp.1206145

## Introduction

Air pollution is associated with impaired lung function growth in childhood and accelerated age-related lung function decline in adulthood (Breton et al. 2011). Mechanisms hypothesized to mediate the association include inflammatory and oxidative stress pathways (Andersen et al. 2011). Local as well as systemic inflammation is associated with poor lung function (Gotz et al. 2011). Persons exposed to inhaled particles from ambient air pollution develop systemic as well as pulmonary inflammation and have systemic oxidative stress, which is characterized by markers in circulating blood, bronchioalveolar lavage, induced sputum, or exhaled breath (Hogg and van Eeden 2009; Sinden and Stockley 2010).

Various markers of obesity such as weight or body mass index (BMI), as well as measures of fat distribution such as waist circumference, ratio of waist circumference to body surface area or height, percentage of fat mass, and skinfold thickness, are also related to both spirometric lung volume and flow parameters, as well as to inflammation in both blood and lung (Salome et al. 2010). The mechanisms by which excess body fat may affect lung function can be categorized as mechanical and nonmechanical (Franssen et al. 2008; Steele et al. 2009).

The mechanical effect of abdominal obesity on lung volumes and associated reductions in airway caliber is thought to be the predominant mode of action (Chen et al. 2007; Salome et al. 2010). This is supported by the observation that the ratio of forced expiratory volume in 1 sec (FEV_1_) to forced vital capacity (FVC) is usually preserved or increased, even in cases of morbid obesity. Both FEV_1_ and FVC decrease in parallel with increasing abdominal obesity, even after controlling for BMI (Hickson et al. 2011). Moreover, obesity stiffens the respiratory system and increases the mechanical work needed for breathing. This is presumed to be attributable to a combination of effects on lung and chest wall compliance (Jones et al. 2007).

Inflammatory pathways may also mediate the influence of obesity on lung function (Probst-Hensch 2010). Effects of obesity on airway caliber and obstruction to air flow that were not merely explained by a mechanical effect on lung volume have been reported in a limited number of studies (e.g., Salome et al. 2010). It is hypothesized that proinflammatory adipokines produced by adipose tissue may contribute to airway remodeling in obese persons (Margretardottir et al. 2009; McClean et al. 2008; Tkacova 2010). Circulating inflammatory markers and adipokines (e.g., soluble tumor necrosis factor receptor 1, adiponectin, leptin) were inconsistently associated with respiratory function in subjects with excess body weight (Lecube et al. 2011; Thyagarajan et al. 2010). Moreover, air pollution and obesity were reported to have greater than additive effects on markers of systemic inflammation among men enrolled in the Normative Aging Study (Madrigano et al. 2009).

In light of inflammatory pathways potentially shared between air pollution and obesity (Chinn et al. 2006; Wise et al. 1998) we investigated whether the association between improved air quality and lung health is also weight dependent. In the SAPALDIA (Swiss Study on Air Pollution and Lung Disease in Adults) cohort, improvements in exposure to PM_10_ (particulate matter with aerodynamic diameter ≤ 10 µm) over an 11-year follow-up period were associated with attenuated age-related lung function decline. The association was strongest for the decline in forced expiratory flow at 25–75% (FEF_25–75_), an early marker of damage to the airways, but was also evident for FEV_1_ and FEV_1_/FVC (Downs et al. 2007). We now investigate whether these previously reported attenuations were modified by BMI.

## Methods

*Study population.* SAPALDIA is a population-based study of the long-term effect of air pollution on respiratory health in the Swiss adult population, as previously described in detail (Ackermann-Liebrich et al. 1997, 2005; Downs et al. 2007; Martin et al. 1997). Briefly, the study comprised eight study areas (Basel, Geneva, Davos, Aarau, Payenne, Montana, Wald, and Lugano) that represent a broad range of geography, climate, urbanization, and air pollution. At baseline in 1991, random population samples of persons 18–60 years of age were invited to participate in SAPALDIA. A total of 8,047 of 9,651 baseline participants (response rate, 83.4%) completed a follow-up assessment in 2002. Valid spirometry data from both surveys were available for 5,741 participants, of whom 4,730 had complete information on BMI and covariates. After excluding 66 subjects, we restricted the present analysis to 4,664 participants (see Supplemental Material, Figure S1).

Ethical approval was obtained from the central ethics committee of the Swiss Academy of Medical Sciences and from the Cantonal Ethics Committees in each of the eight examination areas. All study participants provided written and informed consent before the health examinations.

*Lung function assessment.* In the SAPALDIA study, lung function measurements were conducted using the same spirometer, software, and protocols in both 1991 and 2002 (Sensor Medics 2200SP; Sensor Medics, Yorba Linda, CA, USA). The protocol for the lung function measurements was in accordance with American Thoracic Society (ATS) recommendations (ATS 1991).

Three to eight maneuvers were performed under direction of trained technicians, to comply with ATS acceptability and reproducibility criteria. Lung function parameters used in the present analysis were the highest FEV_1_; FVC; the best peak expiratory flow (PEF); forced expiratory flows at 25%, 50%, and 75% of FVC (FEF_25_, FEF_50_, FEF_75_); and mid-expiratory flow (FEF_25–75_) derived from the best maneuver (defined as the one with the highest sum of FEV_1_ + FVC). All measuring instruments were calibrated at least once a day. Bronchodilation was not conducted. The annual rate of change in lung function (Δlung function/years) was defined for each participant as lung function at follow-up minus lung function at baseline divided by years of follow-up, such that negative values represent a decline in the respective lung function parameters over time.

*Assessment of individual air pollution exposure.* Details of individual assignment of annually averaged home outdoor air pollution exposures are described elsewhere (Downs et al. 2007; Liu et al. 2007). As in the previous studies, concentrations of ambient PM_10_ were used as markers of air pollution. Briefly, we used dispersion models developed by the Swiss Agency for Environment, Forests and Landscape (PolluMap, version 2.0; Swiss Agency for the Environment, Forests, and Landscape, Bern, Switzerland) to estimate each participant’s annual exposure to PM_10_ outside the residence (Ackermann-Liebrich et al. 2005). Inputs for the 1990 and 2000 PolluMap models were hourly meteorological and pollutant emission data from different sources, distributed over 200 × 200 m grid cells. The emission strengths were modeled for diurnal variation, weekday–weekend differences, and seasonal variations. Hourly predictions were averaged over the year, to obtain annual averages for each grid cell. Historical trends of central-site PM_10_ concentrations were used to interpolate values between 1990 and 2000 and extrapolate up to 2003 (Liu et al. 2007). Each participant’s home address was geocoded and assigned to an annual concentration, after the address codes were matched with the concentration grid cell generated by the dispersion models. For the current analysis, we used the difference in the annual average PM_10_ exposures between 2002 and 1991 (ΔPM_10_); thus, a negative value indicates an improvement in air quality.

*Assessment of obesity.* Height was measured (without shoes) in the first and second assessment. Weight was self-reported at baseline and measured (without shoes and coat) at follow-up. We used the BMI (kilograms divided by meters squared) as an obesity parameter. Change in BMI (ΔBMI) was expressed as BMI at follow-up minus BMI at baseline, with positive values reflecting weight gain during follow-up. BMI values at baseline and follow-up were averaged and categorized as underweight (BMI < 18.5), normal weight (BMI ≥ 18.5 to < 25), overweight (BMI ≥ 25 to < 30), and obese (BMI ≥ 30). The average BMI was *a priori* selected over ΔBMI as a primary effect modifier of interest, to better reflect chronic long-term exposure to adipose tissue inflammation.

*Risk factor assessment.* Information on age, sex, smoking status, passive smoking, current and past occupational exposure to dust and fumes, level of education, and hay fever were obtained from self-reported questionnaire data provided at both study examinations. Educational levels were classified into three categories (< 10 years, 10 years, or > 10 years of education) and used as a proxy for socioeconomic status. Participants were classified as atopic if a wheal of at least 3 mm diameter developed in response to one or more of the eight inhalant allergens tested by skin-prick tests at baseline in 1991 (cat, timothy grass, parietaria, birch, house-dust mite, *Alternaria tenuis*, *Cladosporium herbarum*, and dog).

*Statistical analysis.* To assess the modifying effects of average BMI on the association between ΔPM_10_ and Δlung function, we used covariate-adjusted mixed linear models as developed previously for assessing the effect of improved air quality (Downs et al. 2007). The annual rate of decline in lung function was regressed on ΔPM_10_ and average BMI. The adjusted models included the following covariates: ΔBMI, square of average BMI, baseline PM_10_, age, age squared, sex, height, smoking status at baseline (never, former, current), pack-years smoked up to and since baseline, cigarettes smoked per day at baseline and follow-up, passive smoking in childhood, level of education at baseline and change of education, nationality (Swiss or other), presence or absence of occupational exposure to dust or fumes at both examinations, presence or absence of atopy, and seasonal effects (sine and cosine function of day of examination) at both examinations. The models were further adjusted for residual clustering within areas using a random intercept.

Estimated independent associations (and their 95% CIs) of longitudinal changes in lung function parameters with changes in PM_10_ exposure and with average BMI were derived from regression models that included interaction terms between air pollution and average BMI. The hypothesis of average BMI modifying the ΔPM_10_ effect was tested using an interaction term between these two parameters. BMI category–specific effect estimates for ΔPM_10_ were derived from four different models, in which BMI was alternatively centered at each of the mean values of the four BMI categories. Sixty-six observations with a Cook’s distance above the 99.5th percentile in at least one of the basic models for the different lung function parameters were excluded from analysis.

Sensitivity analyses assessed additional ΔPM_10_ interactions with (average BMI)^2^ and ΔBMI. Furthermore, we estimated ΔPM_10_ effects and interactions with average BMI, stratified by sex, by smoking status, by age, and by the presence or absence of a self-report of physician-diagnosed asthma. *p*-Values < 0.05 were interpreted as statistically significant for both main effects and interactions. Statistical analyses were performed using SAS, version 9.2 (SAS Institute Inc., Cary, NC, USA).

## Results

The baseline, follow-up, and change in characteristics of study participants are shown in Table 1. SAPALDIA participants included in the present study were more likely than nonparticipants to be females and never-smokers, and less likely to be of lower social class or overweight (see Supplemental Material, Table S1). Average BMI was classified as normal for most participants (56.3%), whereas 32.8% were obese, and only 17.9% were underweight (Table 1). On average, participants in all BMI categories at baseline gained weight during follow-up.

**Table 1:** Characteristics of study participants (*n* = 4,664) at baseline (SAPALDIA 1) and follow-up (SAPALDIA 2) and change in lung function, BMI, and individually assigned air quality estimates from SAPALDIA 1 to 2.

Characteristic	SAPALDIA 1	SAPALDIA 2	Change SAP1–SAP2
Female [*n* (%)]	2,518 (54)	2,518 (54)
Age (years)	41.3 ± 11.2	52.2 ± 11.2
Height (cm)	169.1 ± 8.8	168.7 ± 8.9
Weight (kg)	67.9 ± 12.5	73.5 ± 14.5
PM_10_ (μg/m^3^)
Median	25.7	20.7	–5.3
IQR	21.6 to 32.3	17.2 to 25.4	–7.6 to –4.2
FVC (mL)	4,487 ± 1,013	4,221 ± 1,014	–266 ± 423
FEV_1_ (mL)	3,541 ± 815	3,157 ± 809	–384 ± 314
FEF_25–75_ (mL/sec)	3,396 ± 1,200	2,624 ± 1,121	–772 ± 684
FEV_1_/FVC (%)	79.2 ± 7.4	74.8 ± 7.3	–4.4 ± 5.1
FEF_25–75_/FVC (%/sec)	76.8 ± 24.9	62.4 ± 23.1	–14.4 ± 17.4
BMI (kg/m^2^)	23.6 ± 3.6	25.7 ± 4.3	2.1 ± 2.2
Average BMI (kg/m^2^)
<18.5 (*n*=80)		17.9 ± 0.58
18.5–<25 (*n*=2,625)		22.3 ± 1.70
25–<30 (*n*=1,549)		27.0 ± 1.41
≥30 (*n*=410)		32.8 ± 2.54
Smoking status (%)
Never	49.3	48.1
Former	20.5	29.0
Current	30.2	22.9
No. of pack-years for ever-smokers
Median	13.9	18.4
IQR	5.2–27.0	7.3–36
No. of cigarettes per day for current smokers
Median	20	15
IQR	10–25	6–20
Passive smoking during childhood (%)	54.0	—^*a*^
Workplace exposure to dust/gases/fumes (%)	30.0	26.8
Swiss nationality (%)	87.7	—^*a*^
Education level (%)^*b*^
Low	13.4	5.9
Intermediate	69.5	66.5
High	17.1	27.6
Increase in education levels between surveys (%)		17.7
Atopy in 1991 (%)^*c*^	21.9
Physician-diagnosed asthma	7.3	7.8
Area (%)
Basel	11.9	11.8
Wald	19.6	19.7
Davos	7.7	7.5
Lugano	14.1	14.2
Montana	9.7	9.6
Payerne	14.1	14.2
Aarau	15.3	15.3
Geneva	7.6	7.7
Values are mean ± SD unless otherwise indicated. ^***a***^Missing values were assessed only at baseline. ^***b***^For the assessment of socioeconomic stsatus the educational level at baseline and the change of educational level between the surveys was assessed. Low education corresponds to primary school level, intermediate to secondary, middle, or apprenticeship school, and high education corresponds to technical college or university. ^***c***^Atopy assessed in 1991, by a skin prick test. Participants were classified as having atopy if they developed response to one or more of the eight inhalant allergens tested (cat, timothy grass, parietaria, birch, house-dust mite, *Alternaria tenuis*, *Cladosporium herbarum*, and dog).			

Table 2 shows the mean annual decline for the different lung function parameters according to average BMI category. Except for FVC, the decline of lung function increased with decreasing average BMI.

**Table 2:** Adjusted mean annual rates of change (95% CI) for the different lung function variables according to average BMI.

Outcome	BMI (kg/m^2^)			
	<18.5	18.5–<25	25–<30	≥30
∆FEV_1_/years (mL/year)	–35.1 (–45.4, –24.8)	–35.9 (–45.8, –6.0)	–35.7 (–45.6, –25.7)	–33.9 (–44.1, –23.8)
∆FVC/years (mL/year)	–15.0 (–27.2, –2.9)	–22.0 (–33.6, –10.5)	–27.9 (–39.5, –16.4)	–37.0 (–44.4, –20.6)
∆FEV_1_/FVC/years (%/year)	–0.6 (–0.7, –0.4)	–0.5 (–0.6, –0.3)	–0.3 (–0.5, –0.3)	–0.2 (–0.4, –0.1)
∆FEF_25–75_/years (mL/sec/year)	–81.9 (–102, –61.6)	–75.7 (–95.0, –56.5)	–67.0 (–86.3, –47.7)	53.9 (–73.8, –33.9)
∆FEF_25–75_/FVC/years (%/sec/year)	–1.8 (–2.3, –1.3)	–1.5 (–1.9, –1.0)	–1.2 (–1.6, –0.7)	–0.8 (–1.3, –0.3)
Estimates are derived from models adjusted for PM_10_ baseline, BMI average, BMI average squared, BMI difference, age, age squared, height, smoking status, pack-years (baseline and follow-up), cigarettes per day, passive smoking during childhood, educational level, workplace exposure, presence of atopy, nationality, season of examination. To compute covariate-adjusted means of lung function decline for the different categories of average BMI, all covariates other than average BMI were centered at their mean values in the sample (*n* = 4,664). The variable BMI average was successively centered at its mean values in the four categories defined by the cutoffs 18.5kg/m^2^, 25kg/m^2^, and 30kg/m^2^. In this way, the adjusted means of interest were provided by the intercept estimates. Negative values indicate decline in lung function between baseline and follow-up examination.				

**Figure 1:**
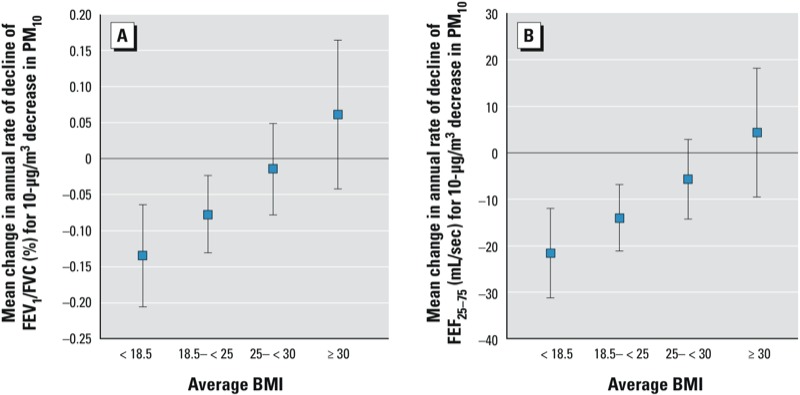
Estimated reduction in average annual lung function decline (95% CI) associated with a 10‑µg/m^3^ decrease in PM_10_ during follow-up for FEV_1_/FVC (*A*) and FEF_25–75_ (*B*) according to average BMI. Estimates are adjusted for PM_10_ baseline, BMI average, BMI average squared, BMI difference, age, age squared, height, smoking status, pack-years (baseline and follow-up), cigarettes per day, parental smoking, educational level, workplace exposure, presence of atopy, nationality, seasonality. Negative estimates indicate a reduction in age-related lung function decline in association with a decrease in PM_10_. Average BMI categories are underweight (< 18.5 kg/m^2^), normal weight (18.5–< 25), overweight (25–< 30), and obese (≥ 30 kg/m^2^).

The median PM_10_ concentration at follow-up was 5.3 µg/m^3^ less than the median concentration at baseline, with an interquartile range (IQR) for the change in PM_10_ of –4.2 to –7.6 µg/m^3^. The improvement in air quality was greater for participants living in urban areas compared with residents of the Alpine regions (Liu et al. 2007). As previously reported (Downs et al. 2007), a decrease in PM_10_ exposure during follow-up was associated with an attenuation of the age-related decline in FEV_1_, FEV_1_/FVC, and FEF_25–75_, but not FVC. The attenuation was strongest for FEF_25–75_ (data not shown). Table 3 presents the association between ΔPM_10_ and lung function decline according to categories of average BMI, expressed as percent attenuation of mean annual decline in lung function (see Table 3 and Figure 1 for estimates expressed as absolute excess decline). Statistically significant interactions between ΔPM_10_ and average BMI were observed for all lung function parameters except FEV_1_. Unexpectedly, improved air quality was associated with a significant acceleration in FVC decline among participants in the lowest average BMI category. In contrast, improved air quality was associated with greater reductions in the annual rates decline of FEF_25–75_, FEF_25–75_/FVC, and FEV_1_/FVC among participants with low or normal average BMI, whereas there was little or no evidence of a beneficial effect of improved air quality on lung function decline among those who were overweight or obese (Table 3, Figure 1; see also Supplemental Material, Table S2). Thus, our findings suggest that beneficial effects of improved air quality on lung function parameters were greatest for participants with a low or normal BMI. The strongest associations between a 10-µg/m^3^ decrease in PM_10_ and a reduction in lung function decline were estimated for the ratio FEF_25–75_/ FVC among those with low and normal average BMI (annual rate of decline reduced by approximately 30% and 22%, respectively) (Table 3).

**Table 3:** Adjusted estimates of the association between change in PM_10_ during follow-up and the annual rates of decline of the different lung function variables (95% CI), according to average BMI and attenuation of decline in lung function parameters associated with a 10‑μg/m^3^ decrease in PM_10_, expressed as a percentage of the mean annual decline for different values of average BMI in all subjects (*n* = 4,664).

Outcome	Estimate	BMI (kg/m^2^)				*p*-Value for interaction
		<18.5	18.5–<25	25–<30	≥30	
∆FEV_1_/years (mL/year)	ΔPM_10_ effect estimate	–2.38 (–6.55, 1.80)	–2.37 (–5.50, 0.75)	–2.37 (–6.11, 1.37)	–2.37 (–8.40, 3.67)	0.99
	Attenuation by ΔPM_10_ (%)	6.8	6.6	6.6	7.0
∆FVC/years (mL/year)	ΔPM_10_ effect estimate	5.64 (0.07, 11.2)	2.10 (–2.07, 6.27)	–1.78 (–6.77, 3.22)	–6.42 (–14.5, 1.65)	0.027
	Attenuation by ΔPM_10_ (%)	–37.6	–9.5	6.4	19.8
FEV_1_/FVC/years (%/year)	ΔPM_10_ effect estimate	–0.14 (–0.21, –0.06)	–0.08 (–0.13, –0.02)	–0.02 (–0.08, 0.05)	0.06 (–0.04, 0.16)	0.005
	Attenuation by ΔPM_10_ (%)	22.5	15.6	5.0	–30.5
∆FEF_25–75_/years (mL/sec/year)	ΔPM_10_ effect estimate	–21.6 (–31.2, –12.0)	–14.0 (–21.1, –6.8)	–5.6 (–14.2, 3.0)	4.4 (–9.5, 18.2)	0.006
	Attenuation by ΔPM_10_ (%)	26.4	18.5	8.4	–8.1
∆FEF_25–75_/FVC/years (%/sec/year)	ΔPM_10_ effect estimate	–0.53 (–0.78, –0.29)	–0.33 (–0.51, –0.14)	–0.10 (–0.32, 0.12)	0.16 (–0.19, 0.52)	0.004
	Attenuation by ΔPM_10_ (%)	29.6	21.8	8.6	–20.6
Effect estimates for a 10-μg/m^3^ change in PM_10_ were computed for the mean BMI values of the respective categories. Estimates are adjusted for PM_10_ baseline, BMI average, BMI average squared, BMI difference, age, age squared, height, smoking status, pack-years (baseline and follow-up), cigarettes per day, parental smoking, educational level, workplace exposure, presence of atopy, nationality, seasonality. Negative estimates indicate a reduction in age-related lung function decline in association with a decrease in PM_10_. Positive ­values in attenuation of decline in lung function indicate a beneficial effect of declining PM_10_ levels (% of mean decline of lung function).						

**Figure 2:**
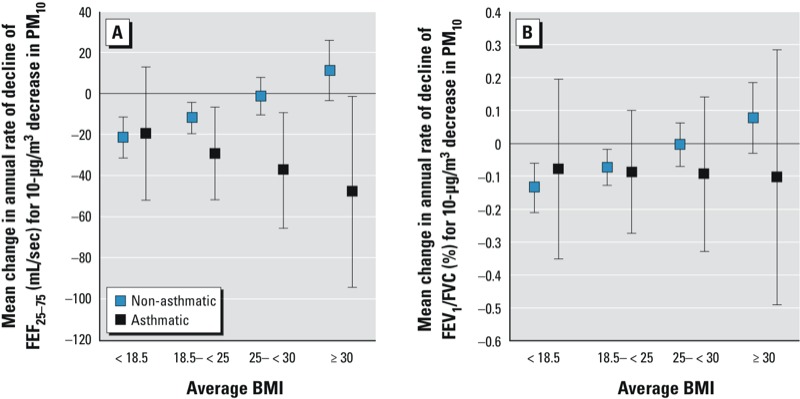
Comparison of the associations between change in PM_10_ during follow-up and the annual changes in the lung function parameters FEV_1_1/FVC and FEF_25–75_ in subjects with and without physician-diagnosed asthma ever, for different values of average BMI (kg/m^2^). Negative estimates indicate a reduction in age-related lung function decline in association with a decrease in PM_10_.

Sensitivity analyses did not indicate interactions of ΔPM_10_ with (average BMI)^2^ or ΔBMI, or differences in effect modification of associations between ΔPM_10_ and lung function decline by BMI according to either sex or smoking (never- vs. ever-smokers) (data not shown). When the analysis was restricted to subjects ≥ 30 years of age, the associations were not materially altered (Table 3). Interestingly, the attenuating association of improved air quality on FEF_25–75_, FEF_25–75_/FVC (see Supplemental Material, Figure S2), and FEV_1_/FVC decline was less dependent on BMI in the subgroup of asthmatics (Figure 2).

## Discussion

This study suggests that attenuation of age-related lung function decline due to improved air quality may be observable only in normal-weight and underweight persons.

The presence of excess weight (as in overweight or obese persons) leads to a mechanical stiffening of the respiratory system. The observed associations between respiratory system stiffening and decreased lung compliance have been attributed to a combination of increased pulmonary blood volume, closure of dependent airways, or increased alveolar surface tension (Salome et al. 2010). Our longitudinal analysis provides novel evidence that the rate of age-related loss in FEF_25–75_ and FEF_25–75_/FVC is slower in obese adults than in normal-weight or underweight adults. This is consistent with the observation that the FEV_1_/FVC ratio is usually normal in obese persons (Salome et al. 2010).

Against this background, several scenarios could explain the observed modification of the association between change in PM_10_ and lung function decline by obesity.

First, a decrease in lung compliance due to weight increase could mask any improvement in small airway function in response to reduced exposure to air pollution. In addition, chronic low-grade inflammation, which is associated with obesity, may limit beneficial effects of improved air quality on peripheral lung tissue. Air pollution exposure and multiple adipokines both have been associated with altered cell proliferation and airway or tissue remodeling (Anderson et al. 2013; Ferecatu et al. 2010; Medoff et al. 2009). The small airway epithelium is thought to play a particularly important role in airway obstruction and accelerated lung function decline in patients with chronic obstructive pulmonary disease (COPD) and asthma, and may contribute to the association of these respiratory diseases with chronic inflammation in response to exposure to particles from tobacco smoke and air pollution (Burgel 2011). The airway remodeling processes induced by chronic inflammation are thought to differ between asthma and COPD patients. In COPD, remodeling of the small airways and lung parenchyma contributes to obstructions in air flow (Sköld 2010), whereas in asthma, airway obstruction may originate predominantly in the larger airways. The role of airway remodeling in modifying lung function response to improved air quality is supported by our previous finding of an interaction between ΔPM_10_ and polymorphisms in apoptosis-related gene (Imboden et al. 2009), as well as by the fact that the observed interactions in this study were restricted to average BMI, which is likely to reflect chronic effects in ΔBMI.

Second, the results may be interpreted as obesity reducing associations between lung function decline and reduction in air pollution. This adverse effect is unlikely direct or causal. Rather, it reflects the obesity paradox in which overweight persons of advanced age have a better prognosis for various chronic conditions, including pulmonary disease (Blum et al. 2011). Although the obesity paradox generally applies to patients rather than to population-based cohorts, we cannot exclude the possibility that excess weight, particularly in the elderly, may reflect a general state of well-being, and thus potentially reduced susceptibility to inflammatory agents. It is possible that because the rate of age-related decline is slower in obese participants, it is not possible to observe a benefit of reduced air pollution on the decline with age.

Unfortunately, in SAPALDIA we had only self-reported weights at baseline, which might be a source of bias due to misclassification. However, all individuals were weighed at the follow-up assessment, and most individuals did not show unexpectedly large weight differences between the two studies. Moreover, weight reporting errors are unlikely to be correlated with air pollution levels and changes. The observation that the interaction between ΔPM_10_ and average BMI appeared to be restricted to nonasthmatics is an exploratory finding and needs confirmation. Independent data on the association of air pollution with lung function and obesity in specific population subgroups is generally sparse. A longitudinal study among persons with asthma found that the adverse effect of weight gain on lung function might be greater for subjects with asthma than for subjects without airflow obstruction (Marcon et al. 2009). In a cross-sectional study of asthma patients, participants who were obese had lower FEV_1_ than their normal-weight counterparts (Pakhale et al. 2010). In a randomized trial of supervised weight loss in 38 obese subjects with asthma, asthma symptoms decreased and lung function improved following weight loss in the treatment group (Stenius-Aarniala et al. 2000). However, none of the studies above evaluated modification of associations between body weight and lung function by air pollution.

A major strength of the present study is its large sample size and the availability of longitudinal data for air pollution exposure, BMI, and lung function after 10 years of follow-up. Lung function measurements were subjected to stringent quality control and conducted by identical devices in all individuals at baseline and follow-up (Künzli et al. 1995, 2005). In previous analyses, we have found associations with air pollution and their modification by genetic factors to be strongest for mid-flow parameters (i.e., FEF_25–75_ and FEF_25–75_/FVC) and consider the availability of these parameters to be of great importance (Chinn et al. 2005). Mid-flow parameters are more sensitive than FEV_1_, and their association with PM_10_ is stronger (Downs et al. 2007; Imboden et al. 2009). They are most commonly used to indicate small airway diseases. Moreover, the authors of a study on FEF_25–75_ and its FVC ratio in families with severe, early-onset COPD reported that these parameters had a high heritability, and suggested that they may be important intermediate phenotypes to consider in genetic linkage and association studies of COPD (DeMeo et al. 2004). Further, it has been shown that small airway inflammatory reactions that result from PM exposures usually occur before the development of tissue destruction and fibrosis and clinically detectable COPD (Niewoehner et al. 1974; Saetta et al. 2001).

## Conclusion

The relationship between obesity, lung function, and air pollution is highly complex. Longitudinal research with additional information on visceral fat and markers of local and systemic inflammation (Parameswaran et al. 2006; Steele et al. 2009) is needed to clarify whether the lung function of obese persons does not benefit from improved air quality, or whether a benefit in the small airways is merely masked.

## Appendix 1: Acknowledgement

We thank the whole SAPALDIA team for their contribution to the study. Additionally, the study could not have been done without the help of the study participants, technical and administrative support, and the medical teams and fieldworkers at the local study sites.

The SAPALDIA team

Study directorate: T. Rochat, J.M. Gaspoz, N. Künzli, N.M. Probst Hensch, C. Schindler.

Scientific team: J.C. Barthélémy, W. Berger, R. Bettschart, A. Bircher, G. Bolognini, O. Brändli, C. Brombach, M. Brutsche, L. Burdet, M. Frey, U. Frey, M.W. Gerbase, D. Gold, E. de Groot, W. Karrer, R. Keller, B. Knöpfli, B. Martin, D. Miedinger, U. Neu, L. Nicod, M. Pons, F. Roche, T. Rothe, E. Russi, P. Schmid-Grendelmeyer, M. Tamm, A. Schmidt-Trucksäss, A. Turk, J. Schwartz, D. Stolz, P. Straehl, J.M. Tschopp, A. von Eckardstein, J.P. Zellweger, E. Zemp Stutz.

Scientific team at coordinating centers: M. Adam, E. Boes, P.O. Bridevaux, D. Carballo, E. Corradi, I. Curjuric, J. Dratva, A. Di Pasquale, L. Grize, D. Keidel, S. Kriemler, A. Kumar, M. Imboden, N. Maire, A. Mehta, F. Meier, H. Phuleria, E. Schaffner, G.A. Thun, A. Ineichen, M. Ragettli, M. Ritter, T. Schikowski, G. Stern, M. Tarantino, M. Tsai, M. Wanner.

Local fieldworkers: Aarau: S. Brun, G. Giger, M. Sperisen, M. Stahel; Basel: C. Bürli, C. Dahler, N. Oertli, I. Harreh, F. Karrer, G. Novicic, N. Wyttenbacher; Davos: A. Saner, P. Senn, R. Winzeler; Geneva: F. Bonfils, B. Blicharz, C. Landolt, J. Rochat; Lugano: S. Boccia, E. Gehrig, M.T. Mandia, G. Solari, B. Viscardi; Montana: A.P. Bieri, C. Darioly, M. Maire; Payerne: F. Ding, P. Danieli, A. Vonnez; Wald: D. Bodmer, E. Hochstrasser, R. Kunz, C. Meier, J. Rakic, U. Schafroth, A. Walder.

Administrative staff: C. Gabriel, R. Gutknecht.

## Supplemental Material

(2.6 MB) PDF
