# Supplementary material for: Improved Air Quality and Attenuated Lung Function Decline: Modification by Obesity in the SAPALDIA Cohort
Source: Environ Health Perspect. 2013 Jul 2;121(9):1034–9. doi: 10.1289/ehp.1206145 (PMC3764076; doi:10.1289/ehp.1206145)
Supplement: (2.6 MB) PDF [file ehp.1206145.s001.pdf]

Supplemental Material

Improved Air Quality and Attenuated Lung Function Decline: Modification by Obesity in the SAPALDIA Cohort

Tamara Schikowski, Emmanuel Schaffner, Flurina Meier, Harish C. Phuleria, Andrea Vierkötter, Christian Schindler, Susi Kriemler, Elisabeth Zemp, Ursula Krämer, Pierre-Olivier Bridevaux, Thierry Rochat, Joel Schwartz, Nino Künzli, and Nicole Probst-Hensch

Table of contents

Supplemental Material, Figure S1. Flowchart showing the SAPALDIA study population from baseline to follow up……………………………………………………………….... Page 2

Supplemental Material, Table S1. Comparison of characteristics at baseline and follow-up for subjects Included versus excluded from the analyses…………………………………..Page 3

Supplemental Material, Table S2. Adjusted estimates of the association of change in PM10 during follow-up and the annual rates of decline of the different lung function variables, for different values of average BMI for all subjects and for subjects > 30 years of age………Page 6

Supplemental Material, Figure S2. Comparison of the associations between change in PM10 during follow-up and the annual changes in the lung function parameters FEV1, FVC and FEF25-75/FVC in subjects with and without physician diagnosed ever asthma…………..Page 7

Supplemental Material, Table S1. Comparison of characteristics at baseline and follow-up for subjects included versus excluded from the analyses.

Variables	Included N=4664	Not included	P value Included vs. not included	Total N available in testing included vs. excluded
Female Sex (%)	54	48	<0.001	9651
Age at Baseline	41.3±11.2	40.9±12.0	0.09	9651
Age at follow-up	52.2±11.2	52.0±12.0	0.4	8047
Height [cm]	169.1±8.8	169.3±9.3	0.3	9552
Height at follow-up [cm]	168.7±8.9	169.6±9.7	<0.001	6601
Weight [kg]	67.9±12.5	69.8±13.8	<0.001	9552
Weight at follow-up [kg]	73.5±14.5	75.8±15.8	<0.001	6598
BMI at baseline [kg/m^2]	23.6±3.6	24.3±4.1	<0.001	9552
BMI at follow-up [kg/m^2]	25.7±4.3	26.3±4.7	<0.001	6598
BMI baseline – follow-up [kg/m^2]	2.1±2.2	2.0±2.4	0.11	6568
**Average BMI**				
<18.5	17.9±0.6	17.8±0.5	0.69	105
18.5 – <25	22.3±1.7	22.4±1.7	0.04	3606
25 – <30	27.0±1.4	27.1±1.4	0.54	2234
≥30	32.7±2.5	33.3±3.3	0.02	623
**Smoking status at baseline (%)**				
Never smoker	49.3	38.8	<0.001	9636
Ex-smoker	20.5	24.5	<0.001	9636
Current smoker	30.2	36.7	<0.001	9636
**Smoking status at follow up (%)**				
Never smoker	48.1	34.1	<0.001	7612
Ex-smoker	29.0	34.1	<0.001	7612
Current smoker	22.9	31.8	<0.001	7612
**No. of pack-years for ever smokers**				
Median at baseline	13.9	14.0	0.6	5226
Median at follow-up	18.4	18.0	0.14	3780
**Number of cigarettes per day for current smoker**				
Median at baseline	20	20	0.65	2314
Median at follow-up	15	13	<0.001	2000
Passive smoking during childhood (%)	54.0	55.9	0.06	9651
Workplace exposure to dust/gases/fumes at baseline (%)	30.0	33.3	<0.001	9620

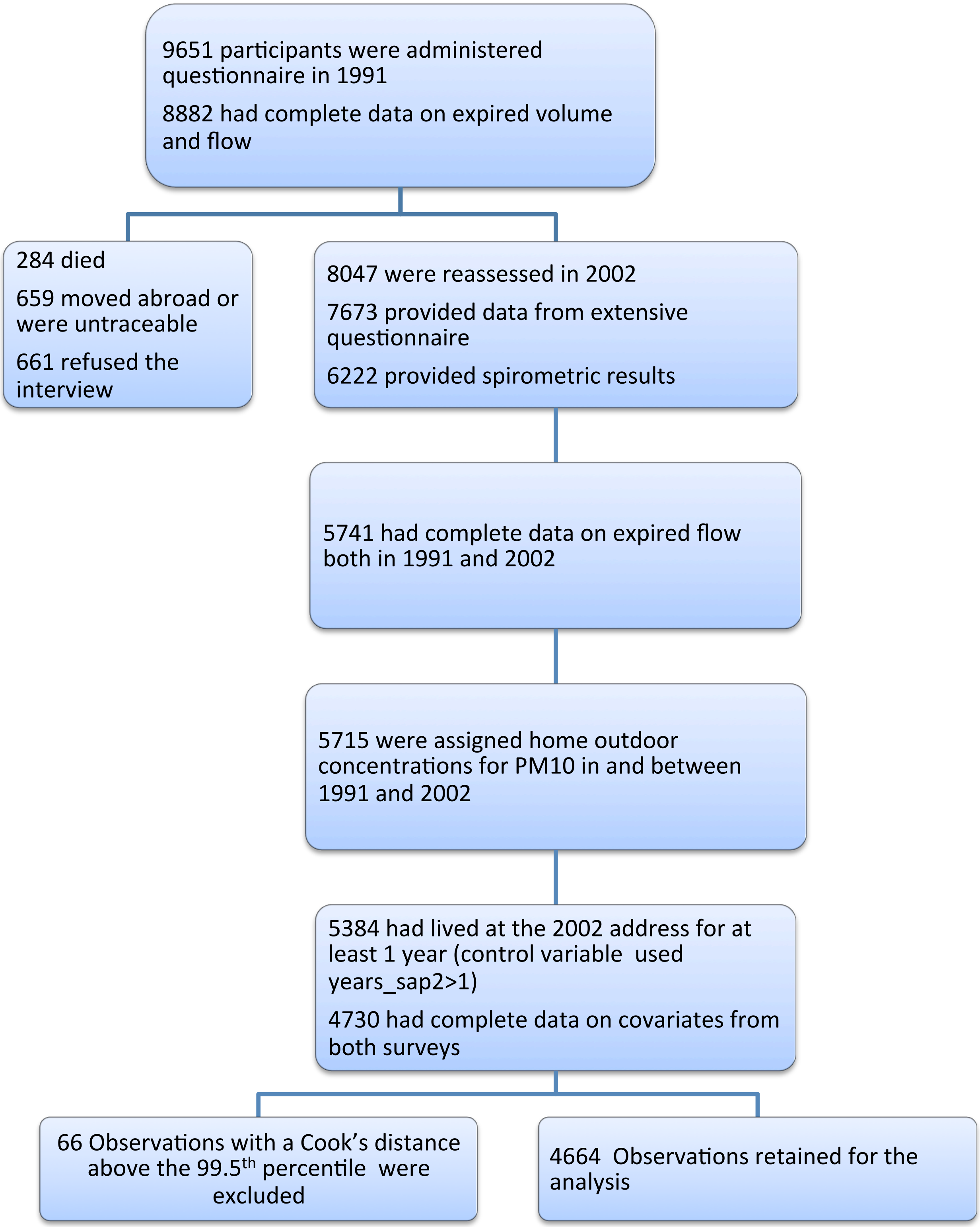

Supplemental Material, Figure S1. Flowchart showing the SAPALDIA study population from baseline to follow up.

Variables	Included N=4664	Not included	P value Included vs. not included	Total N available in testing included vs. excluded
Workplace exposure to dust/gases/fumes at follow-up (%)	26.8	29.1	0.05	6563
**Education level at baseline (%)[a]**				
Low	13.4	20.1	<0.001	9627
Intermediate	69.5	63.6	<0.001	9627
High	17.1	16.3	0.29	9627
Education levels increased between surveys (%)	17.7	11.8	<0.001	7673
Atopy in 1991 (%)[b]	21.9	22.5	0.49	9651
Physician diagnosed asthma at baseline (%)	7.3	8.7	0.01	9644
Physician diagnosed asthma at follow-up (%)	7.8	8.4	0.38	7664
**PM_{10} µg/m^3**				
Median at baseline	25.7	29.9	<0.001	9552
Median at follow-up	20.7	22.7	<0.001	7950
ΔPM_{10} µg/m^3	-5.3	-5.4	0.76	7950
**Mean lung function at baseline**				
FVC (ml)	4487±1013	4469±1061	0.40	9050
FEV_1 (ml)	3541±815	3522±880	0.30	9050
FEF_{25-75} (ml/sec)	3396±1200	3411±1291	0.58	8882
FEV_1/FVC (%)	79.2±7.4	79.1±8.5	0.46	8957
FEF_{25-75}/FVC (%/sec)	76.8±24.9	77.4±27.2	0.27	8882
**Lung function at follow-up**				
FVC (ml)	4221±1015	4332±1092	<0.001	6205
FEV_1 (ml)	3157±809	3271±894	<0.001	6222
FEF_{25-75} (ml/sec)	2624±1121	2862±1203	<0.001	6069
FEV_1/FVC (%)	74.8±7.3	75.4±8.0	0.01	6127
FEF_{25-75}/FVC (%/sec)	62.4±23.1	65.6±24.6	<0.001	6069
**Area at baseline (%)**				9651
Basel	11.9	18.9	<0.001	
Wald	19.6	12.1	<0.001	
Davos	7.7	7.8	0.81	
Lugano	14.1	13.0	0.11	
Montana	9.7	6.9	<0.001	
Payerne	14.1	16.8	<0.001	
Aarau	15.3	11.7	<0.001	
Geneva	7.6	12.9	<0.001	

Values are means ± standard deviation unless otherwise indicated.

Abbreviations: FEF_{25-75} is forced expiratory flow between 25% and 75% of forced vital capacity (FVC). FEV_1 is forced expiratory volume in one second, and PM_{10} is particulate matter with an aerodynamic diameter of less than 10μg

[a] For the assessment of SES the educational level at baseline and the change of educational level between the surveys was assessed.

[b]Atopy assessed in 1991, by a skin prick test. Participants were classified as having atopy if they developed response to one or more of the 8 inhalant allergens tested (cat timothy grass, parietaria, birch, house-dust mite, *Alternaria tenuis*, *Cladosporium herbarum* and dog)

Supplemental Material, Table S2. Adjusted estimates of the association of change in PM_{10} during follow-up and the annual rates of decline of the different lung function variables according to average BMI among participants > 30 years of age (N = 3,788)[a]

	BMI (kg/m^2)				
Outcome	<18.5	18.5 – <25	25 – <30	≥ 30	p-value for interaction[b]
ΔFEV_1/years (ml/y)	-3.73 (-9.20, 1.74)	-2.73 (-6.75, 1.29)	-1.69 (-5.99, 2.61)	-0.43 (-7.05, 6.19)	0.48
ΔFVC/years (ml/y)	3.58 (-3.89, 11.04)	1.02 (-4.48, 6.51)	-1.66 (-7.53, 4.22)	-4.88 (-13.92, 4.17)	0.19
ΔFEV_1/FVC/years (%/y)	-0.12 (-0.22, -0.03)	-0.07 (-0.14, 0.01)	-0.01 (-0.08, 0.07)	0.06 (-0.05, 0.18)	0.02
ΔFEF_{25-75}/years (ml/sec/y)	-19.80 (-32.57, -7.02)	-12.59 (-21.98, -3.19)	-5.06 (-15.11, 4.98)	4.01 (-11.47, 19.48)	0.03
ΔFEF_{25-75}/FVC/years (%/sec/y)	-0.44 (-0.78, -0.11)	-0.28 (-0.53, -0.04)	-0.12 (-0.38, 0.15)	0.09 (-0.32, 0.49)	0.07

[a]Estimate of the average effect of a 10μg/m^3 change in PM_{10} during follow-up on the annual rate of change in the respective lung function parameter, with 95%-confidence interval; negative estimates indicate a beneficial effect of declining PM10-levels. Estimates are adjusted for PM10 baseline, BMI average, BMI average squared, BMI difference, age, age squared, height, smoking status, pack-years (baseline and follow-up), cigarettes per day, parental smoking, educational level, workplace exposure, presence of atopy, nationality, seasonality.

[b] p-value of the interaction term of ΔPM_{10} with average BMI

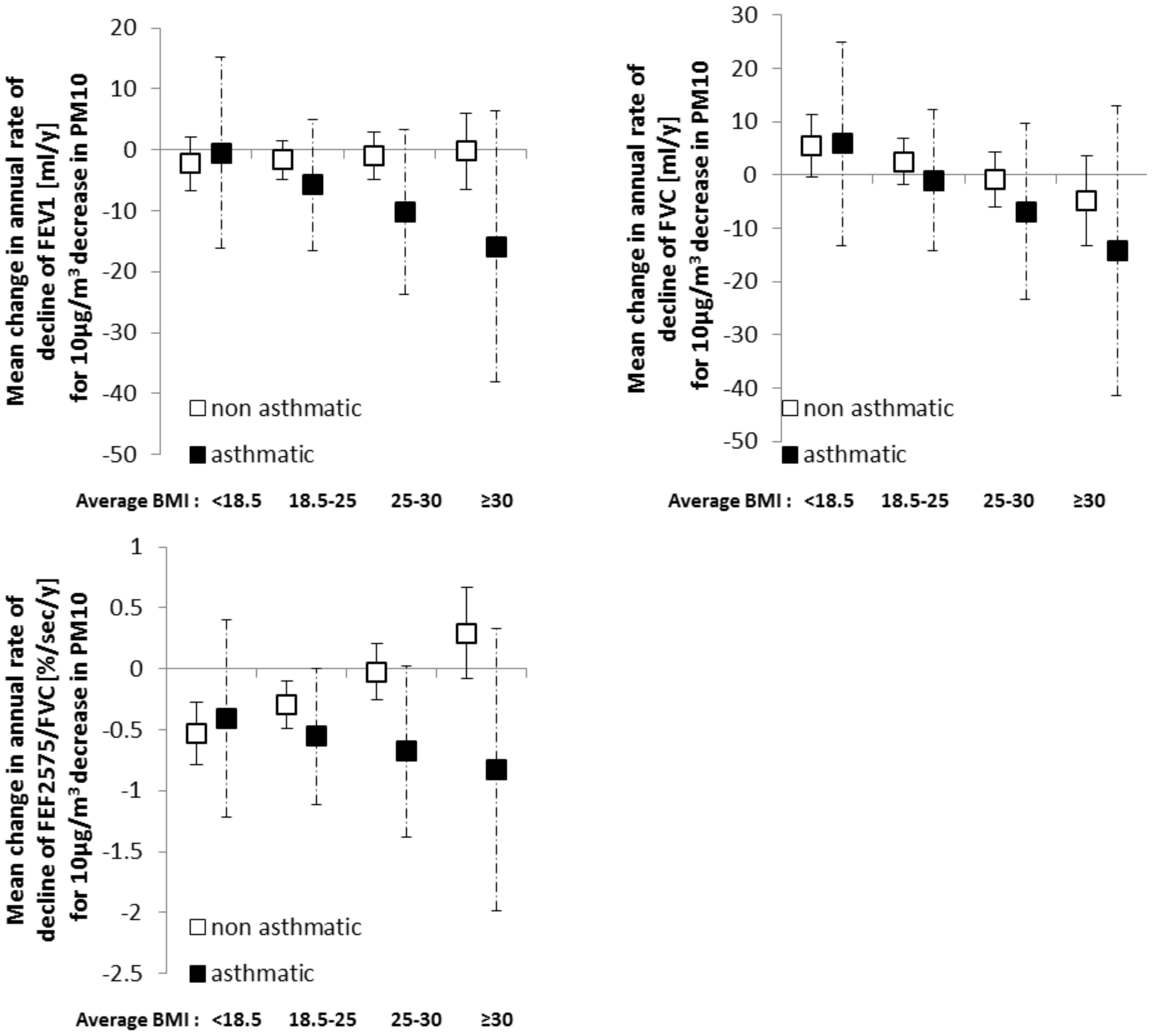

Supplemental Material, Figure S2: Comparison of the associations between change in PM_{10} during follow-up and the annual changes in the lung function parameters FEV_1, FVC, and FEF_{25-75}/FVC in subjects with and without physician diagnosed ever asthma, for different values of average BMI in kg/m^2. Estimates (95%CI) are adjusted for PM_{10} baseline, BMI average, BMI average squared, BMI difference, age, age squared, height, smoking status, pack-years (baseline and follow-up), cigarettes per day, parental smoking, educational level, workplace exposure, presence of atopy, nationality, seasonality. Negative estimates indicate a reduction in age related lung function decline in association with a decrease in PM_{10}.
